# Combinations of Low-Frequency Genetic Variants Might Predispose to Familial Pancreatic Cancer

**DOI:** 10.3390/jpm11070631

**Published:** 2021-07-02

**Authors:** Emily P. Slater, Lisa M. Wilke, Lutz Benedikt Böhm, Konstantin Strauch, Manuel Lutz, Norman Gercke, Elvira Matthäi, Kari Hemminki, Asta Försti, Matthias Schlesner, Nagarajan Paramasivam, Detlef K. Bartsch

**Affiliations:** 1 Department of Visceral, Thoracic and Vascular Surgery, Philipps University, D-35043 Marburg, Germany; Wilke.lisa.marie@gmail.com (L.M.W.); lutz-bene-boehm@web.de (L.B.B.); gercken@staff.uni-marburg.de (N.G.); Elvira.matthaei@med.uni-marburg.de (E.M.); bartsch@med.uni-marburg.de (D.K.B.); 2 Institute of Medical Biometry, Epidemiology and Informatics (IMBEI), University Medical Center, Johannes Gutenberg University, D-55101 Mainz, Germany; strauch@uni-mainz.de (K.S.); manuel.lutz@uni-mainz.de (M.L.); 3 Helmholtz Zentrum München—German Research Center for Environmental Health, Institute of Genetic Epidemiology, D-85764 Neuherberg, Germany; 4 Chair of Genetic Epidemiology, IBE, Faculty of Medicine, LMU Munich, D-81377 Munich, Germany; 5 German Cancer Research Center (DKFZ), Division of Molecular Genetic Epidemiology, D-69120 Heidelberg, Germany; K.Hemminki@dkfz-heidelberg.de (K.H.); A.Foersti@kitz-heidelberg.de (A.F.); 6 German Cancer Research Center (DKFZ), Division of Cancer Epidemiology, D-69120 Heidelberg, Germany; 7 Biomedical Center, Faculty of Medicine and Biomedical Center in Pilsen, Charles University in Prague, 30605 Pilsen, Czech Republic; 8 Hopp Children’s Cancer Center (KiTZ), D-69120 Heidelberg, Germany; 9 German Cancer Research Center (DKFZ), Division of Pediatric Neuro Oncology, German Cancer Consortium (DKTK), D-69120 Heidelberg, Germany; 10 Bioinformatics and Omics Data Analytics, German Cancer Research Center, D-69120 Heidelberg, Germany; matthias.schlesner@informatik.uni-augsburg.de; 11 Biomedical Informatics, Data Mining and Data Analytics, Augsburg University, D-86159 Augsburg, Germany; 12 Computational Oncology, Molecular Diagnostics Program, National Center for Tumor Diseases (NCT), German Cancer Research Center (DKFZ), D-69120 Heidelberg, Germany; n.paramasivam@dkfz-heidelberg.de

**Keywords:** familial pancreatic cancer, WGS, genetic variants

## Abstract

Familial pancreatic cancer (FPC) is an established but rare inherited tumor syndrome that accounts for approximately 5% of pancreatic ductal adenocarcinoma (PDAC) cases. No major causative gene defect has yet been identified, but germline mutations in predisposition genes *BRCA1/2*, *CDKN2A* and *PALB2* could be detected in 10–15% of analyzed families. Thus, the genetic basis of disease susceptibility in the majority of FPC families remains unknown. In an attempt to identify new candidate genes, we performed whole-genome sequencing on affected patients from 15 FPC families, without detecting *BRCA1/2*, *CDKN2A* or *PALB2* mutations, using an Illumina based platform. Annotations from CADD, PolyPhen-2, SIFT, Mutation Taster and PROVEAN were used to assess the potential impact of a variant on the function of a gene. Variants that did not segregate with pancreatic disease in respective families were excluded. Potential predisposing candidate genes *ATM, SUFU*, *DAB1*, *POLQ, FGFBP3,* *MAP3K3* and *ACAD9* were identified in 7 of 15 families. All identified gene mutations segregated with pancreatic disease, but sometimes with incomplete penetrance. An analysis of up to 46 additional FPC families revealed that the identified gene mutations appeared to be unique in most cases, despite a potentially deleterious *ACAD9* Ala326Thr germline variant, which occurred in 4 (8.7%) of 46 FPC families. Notably, affected PDAC patients within a family carried identical germline mutations in up to three different genes, e.g., *DAB1*, *POLQ* and *FGFBP3*. These results support the hypothesis that FPC is a highly heterogeneous polygenetic disease caused by low-frequency or rare variants.

## 1. Introduction

Pancreatic ductal adenocarcinoma (PDAC) is projected to become the second leading cause of cancer-related death in Germany by 2030 [1]. A familial aggregation of PDAC, so-called familial pancreatic cancer (FPC), has been well-established in about 5–10% of cases [2]. An inherited predisposition to PDAC occurs in hereditary tumor predisposition syndromes such as Peutz–Jeghers syndrome (PJS) or hereditary breast and ovarian cancer (HBOC), hereditary pancreatitis and, finally, as familial pancreatic cancer (FPC) syndrome. FPC describes families with at least two first-degree relatives with PDAC that do not fulfill the criteria for another inherited tumor syndrome [3,4]. The inheritance of PDAC is mostly autosomal dominant with a heterogeneous phenotype [5,6]. The identification of disease-causing genes is important to determine the genetic risk for the development of PDAC in an individual from an FPC family since carriers of deleterious germline mutations profit more from early screening, as they have a higher risk compared to individuals from FPC families without a detectable mutation [7]. The screening of mutation carriers and the early detection of PDAC or its high-grade precursor lesions might offer the best chance of reducing the high mortality rates of this disease [8,9]. A major predisposing gene defect has not yet been identified in FPC families, but germline mutations in *BRCA1, BRCA2, CDKN2A, PALB2* and *ATM* were identified in 8% to 16% of FPC families [3,10]. Twenty-five (16.6%) of 150 genetically analyzed FPC families from the German National Case Collection for familial PDAC (FaPaCa) revealed potentially causative germline mutations in the *BRCA1, BRCA2, CDKN2A* and *PALB2* genes [5]. The most frequently mutated gene (6%) was the BRCA2 gene, even in the absence of breast cancer, as already reported by others [4,11,12]. These studies underscore that the genetic basis of FPC remains unclear at present in over 80% of families. However, a North American study reported potential pathogenic, low-frequency germline variants in the *BUB1B,* *CPA1,* *FANCC* and *FANCG* genes in single FPC families, based on whole-genome sequencing of FPC patients without identified germline mutations in the known predisposition genes *BRCA1/2,* *CDKN2A* and *PALB2* [10].

As pointed out, the genetic basis is not known in 80% of the families; therefore, to better understand the basis of inherited cancer susceptibility, we performed whole-genome sequencing (WGS) on affected members from fifteen FPC families without previously detected *BRCA1/2, PALB2* or *CDKN2A* germline mutations to identify additional FPC candidate genes.

## 2. Materials and Methods

### 2.1. Recruitment of Cases and High Risk Individuals

The FaPaCa registry was established as a national case collection for FPC by the Deutsche Krebshilfe in 1999 [13]. Members of FPC families were recruited by direct referral via their physicians or by personal contact with the FaPaCa study office based on information about the study, e.g., via the internet (http://www.fapaca.de, accessed on 31 May 2021), respectively. Information was obtained from probands in all families and was corroborated by all other relatives who agreed to participate. All participants underwent counseling and a family pedigree was constructed based on the personal interview. All cancer diagnoses in the family were confirmed by a review of medical and pathological records, death certificates and by revision of the pathology slides, whenever available. All participants provided written informed consent and the study was approved by the Ethics Committee of the Philipps-University Marburg (No. 36/1997, last amendment 5/2009). The diagnosis of FPC was based on the presence of two or more first-degree relatives with a confirmed diagnosis of PDAC, without fulfilling the criteria of any other inherited tumor syndrome (e.g., HBOC) at the time of inclusion. All probands were Caucasian, with none reporting Ashkenazi Jewish heritage. Affected index patients, who gave informed consent, were offered a mutation analysis of the candidate genes, such as *BRCA2*, *CDKN2A* and *PALB2*. These results were published earlier [14,15,16,17]. For the presented analysis, we chose 15 FPC families that fulfilled the following criteria: at least 3 affected PDAC patients, no identified deleterious *BRCA1/2*, *CDKN2A* or *PALB2* germline mutation in previous testing and available blood DNA from at least two affected and two unaffected individuals. Ideally, DNA was also available from the corresponding PDAC tissue of affected patients.

### 2.2. DNA Extraction

The genomic DNA (gDNA) of participating family members was isolated from peripheral blood leukocytes and/or tumor tissue using the QIAamp DNA kit (Qiagen, Hilden, Germany) according to the manufacturer’s protocol.

### 2.3. Next Generation Sequencing of PDAC Cases, High Risk Individuals and Non-PDAC Cases

Whole-genome sequencing (WGS) for the cases and controls was performed with genomic DNA on Illumina HiSeq at the DKFZ, Heidelberg, Germany. The results of the sequencing were paired-end-reads (read length 150 bp), which were aligned using BWA-MEM (v. 0.7.8) [18]. After alignment, variant calling was performed with the GotCloud variant calling pipeline [19], which is an SVM (support vector machine)-based tool to call and filter genetic variants. After calling, the variants were annotated using ANNOVAR [20], a tool for adding external database data to the corresponding variants. The databases used, dbnsfp33a [21,22] and 1000 g [23], added information about the region of the single-nucleotide variant (SNV). To classify the possible effect of a variant on the phenotype, the following tools were used: CADD, which gives a score regarding the deleteriousness of single-nucleotide variants and insertion/deletion (indel) variants in the human genome (http://cadd.gs.washington.edu/, accessed on 31 May 2020); PolyPhen-2, to evaluate the possible impact on an amino acid substitution on the structure and function of a human protein (http://genetics.bwh.harvard.edu/pph2/index.shtml, accessed on 31 May 2020); SIFT, to analyze the effect of an amino acid substitution on protein function, based on multiple alignments (http://sift.jcvi.org, accessed on 31 May 2020); Mutation Taster, to clarify the disease-causing potential of DNA sequence alterations (http://www.mutationtaster.org, accessed on 31 May 2020; and PROVEAN, to check the impact of an amino acid substitution or indel on the biological function of a protein (http://provean.jcvi.org/index.php, accessed on 31 May 2020), respectively. A total score for each variant was calculated by adding up the ratings of “damaging” or “deleterious” using the tools listed above. Variants with a high total score were considered first. Next, variants in FPC risk genes [10] with CADD scores less than 20 were taken into consideration. Finally, high-scoring variants occurring in multiple families were also considered. These variants were then eliminated if they did not co-segregate with PDAC or its high-grade precursor lesions. The identified co-segregating gene variants from these 15 families were then analyzed in one affected patient from another 15 to 34 independent FPC families by Sanger sequencing, as previously described [17]. If the case showed a variant in the respective gene, then all family members with available blood samples were tested for the variant.

### 2.4. Immunohistochemical Staining

For DAB1 immunostaining, formalin-fixed and paraffin-embedded archived tumor samples and corresponding normal tissues were stained as follows. Paraffin sections (4 µm thickness) were stained using a polyclonal anti-DAB1 antibody (Novus NBP2-16095) and a standard VectaStain Protocol.

## 3. Results

To identify pathogenic or likely pathogenic variants, we focused the WGS analysis on the potentially most informative 15 FPC families of the FaPaCa registry with a total of 57 cases and 59 healthy controls. The median age of affected PDAC patients in these families was 64 (range 42 to 78) years. The initial analyses identified 4683 possibly damaging non-synonymous SNVs. These were first categorized by the total score for damaging or deleterious, as described in the methods section and then eliminated if they did not co-segregate with the PDAC disease. The best candidates were chosen based on their functional prediction score, identification as a risk gene or their occurrence in multiple families. Of the 15 initially tested families, 7 carried potentially causative germline variants in seven genes, and, in 3 families, up to three co-segregating variants in different genes were identified (Table 1). The low-frequency or rare gene variants were identified in the *ATM, SUFU*, *DAB1*, *POLQ*, *FGFBP3, MAP3K3* and *ACAD9* genes.

**Figure 1:**
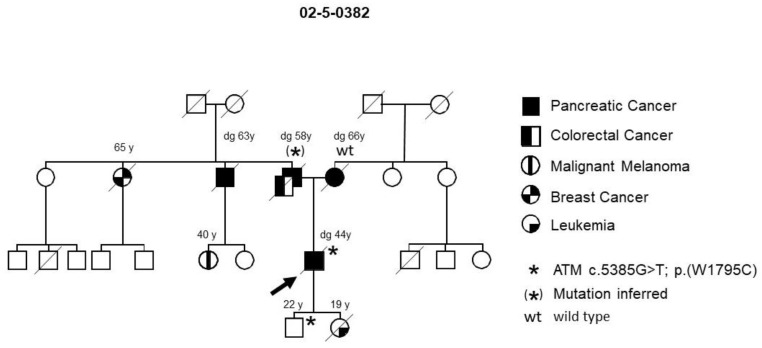
FPC family with an *ATM* germline variant. The arrow designates the index patient. Diagonal lines indicate deceased individuals.

A deleterious variant in the FPC risk gene, *ATM* (*Ataxia telangiectasia mutated*), c.5385G > T; p.W1795C, was identified in one (family 02-5-0382) of the 15 initial families (Figure 1). PDAC tumor tissue from the mother of the index patient was found to be wild type, while blood or tissue from his affected father and uncle was not available, but the 22-year-old son also tested positive, thus supporting segregation with disease. PDAC screening of the son has thus far been uneventful. The daughter of the index patient had a stem cell transplantation and succumbed to leukemia before germline DNA testing was performed. Thus, it could not be determined whether she originally carried the germline *ATM* variant. However, since A*TM* mutations are associated with a high incidence of leukemia in childhood [24], she possibly carried the variant as well. Further validation of this gene showed it to be mutated in one of 36 tested FPC families (2.8%).

**Figure 2:**
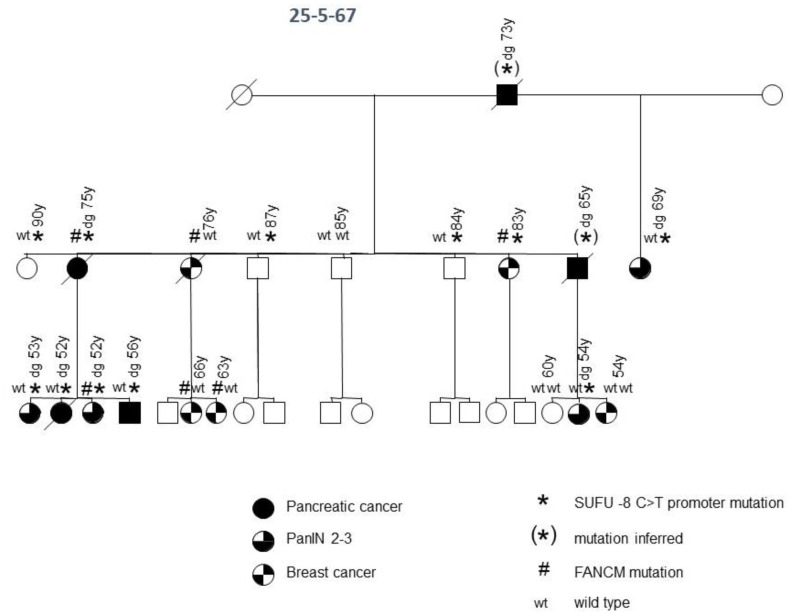
FPC family with *SUFU* and *FANCM* germline variants. Diagonal lines indicate deceased individuals.

In another (family 25-5-67) of the 15 FPC families, a deleterious promoter variant of the *SUFU* gene, a negative regulator of hedgehog signaling on chromosome 10, at position −8 upstream of the start of transcription, was identified (Figure 2). This promoter variant has a CADD score of 20.5 and breaks 470 non-coding elements. A potential binding site for the AP-2 transcription factor would be disrupted by this variant, which could result in a reduction in the expression level of this tumor suppressor. This family was initially included as an FPC family, but, over time, they turned out to be a family with an accumulation of PDAC and breast cancer, since three family members in the third generation developed breast cancer during follow-up. In this family, all three tested patients with PDAC and all four tested patients with high-grade PDAC precursor lesions (pancreatic intraepithelial neoplasia 2/3) carried the variant. Only one of the five tested individuals with breast cancer possessed the variant. Notably, three older (84, 87 and 90 years), yet healthy, individuals also carried the *SUFU* variant. Thus, assuming incomplete penetrance, this variant meets the criteria to be a causative germline mutation for pancreatic neoplasia in this family. None of the other 14 FPC families tested carried a *SUFU* germline variant. Interestingly, this family also carried a germline variant in the *FANCM* gene. The missense variant P1459A tends to be deleterious, but it did not segregate with the pancreatic disease in this family (Figure 2). However, four of five patients with breast cancer tested positive for this variant, whereas none of the individuals without breast cancer carried the variant. Thus, the variant might contribute to the development of breast cancer in this family. Potentially pathogenic variants of *FANCM* were not identified in the other 14 families tested by WGS.

**Figure 3:**
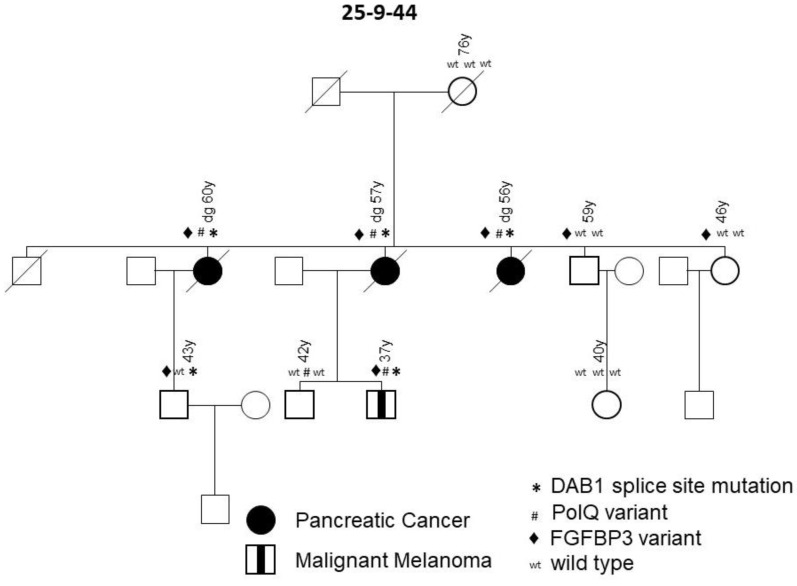
FPC family with variants in *DAB1*, *PolQ* and *FGFBP3* genes. Diagonal lines indicate deceased individuals.

Family 25-9-44 carried a variant in the *disabled-homolog 1* gene on chromosome 1 (*DAB1*, * in Figure 3). This deleterious splice site variant, c.786 + 1G > A, co-segregates with the disease in all affected individuals and was not present in healthy older family members. During follow-up, a melanoma occurred in a mutation carrier in the third generation, meaning that this family meets the criteria of a family with a pancreatic carcinoma/melanoma syndrome (PCMS). To evaluate the role of *DAB1* germline mutations in pancreatic carcinoma/melanoma-prone families and their function with regard to a predisposition to FPC, Sanger sequencing of DNA obtained from the blood of an additional 31 PCMS index patients with either PDAC or malignant melanoma enrolled in the FaPaCa registry was performed. In total, 49 families were tested, including 32 PCMS families and 17 FPC families. The splice site variant in the *DAB1* gene, however, was detected in only one PCMS family (2%).

For expression analyses of DAB1, the tissue of one positive tested index patient was immunohistochemically stained and revealed the normal expression of DAB1 in the epithelium of healthy gallbladder and plasma cells, but a loss of expression in the PDAC lesion (data not shown). DNA obtained from the PDAC of this patient was sequenced and showed the heterozygous splice site variant of a germline nature. The loss of DAB1 protein expression in the tumor tissue compared to healthy tissue suggests a somatic second hit.

The same family, 25-9-44, also carried the P1381T variant in the FPC risk gene, *POLQ* (# in Figure 3). This low-frequency variant, which was scored as a variant of unknown significance, also co-segregated with the PDAC and melanoma. One 42-year-old healthy male with multiple nevi also carried this variant (Figure 3, 42 y), but two older and two younger healthy controls had wild type sequences. Despite being a low-frequency variant, it could play a contributing role in the genesis of the disease. Only one of a total of 29 families screened carried the *POLQ* gene variant. Notably, family 25-9-44 carried a third germline variant in the gene for fibroblast growth factor binding protein 3 (*FGFBP3*) on chromosome 10, namely the missense variant A242T, which was scored as deleterious (♦ in Figure 3). The variant was present in all three PDAC cases, as well as the melanoma patient, and thus also segregated with disease. However, since this variant was also found in two older, yet healthy, controls, it did not show complete penetrance. The variant was only detected in one out of a total of 29 tested FPC families. In summary, all patients affected with PDAC and melanoma in family 25-9-44 carried the *DAB1*, *POLQ* and *FGFBP3* variants, whereas none of the healthy individuals carried all three variants.

**Figure 4:**
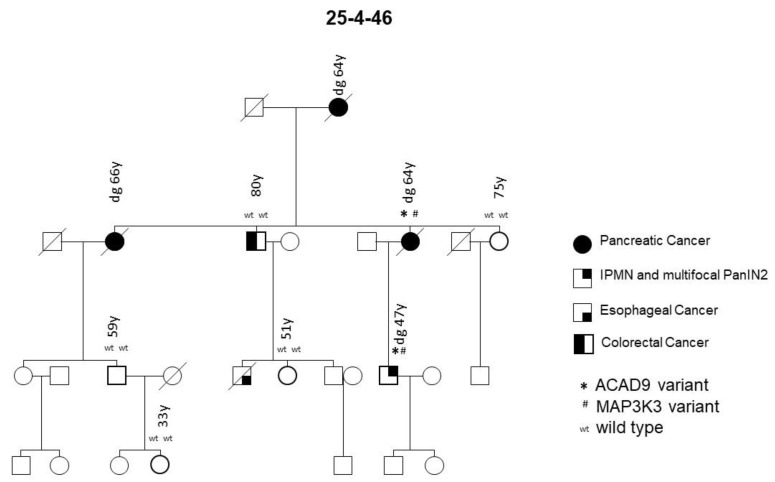
FPC family with *MAP3K3* and *ACAD9* germline variants. Diagonal lines indicate deceased individuals.

Family 25-4-46 had a deleterious missense variant I504T in the *Mitogen-activated Protein Kinase Kinase Kinase 3* (*MAP3K3*) gene on chromosome 17 (Figure 4). This variant was detected in both the tested PDAC case and the case with pancreatic precursor lesions in this family, but not in any of the five family members without PDAC or detected precursor lesions, thus segregating with the disease. Only one of a total of 29 FPC families tested carried this variant.

**Figure 5:**
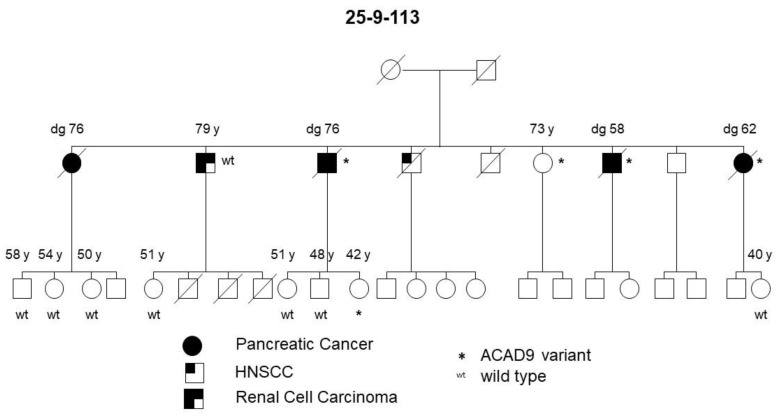
FPC family with a germline *ACAD9* A326T variant. Diagonal lines indicate deceased individuals.

The PDAC and pancreatic precursor lesion patients of family 25-4-46 also carried a potential pathogenic *ACAD9* germline variant (Table 1, Figure 4). *ACAD9* is localized on chromosome 3q21.3. The *ACAD9* germline variant c.976G > A; p.(A326T) was found in three of the initially 15 sequenced families (see Figure 5 for an example). The variant allele carries G > A, resulting in an amino acid change from Ala to Thr at amino acid position 326. This position is highly conserved among species and the entry for this SNV in ClinVar/variation described this germline variant to be of uncertain significance (www.ncbi.nlm.nih.gov/clinvar/variation/136253, accessed on 31 May 2020). In all three families carrying this SNV, the variant co-segregated with disease, since every affected individual with either PDAC or its high-grade precursor lesions (PanIN2/3) had the variant. As none of these families reported breast cancer in the pedigree, affected members of an additional 31 FPC families without breast cancer were Sanger sequenced to determine the status of the *ACAD9* gene. Since one of the additional families carried this SNV, a total of 4 out of 46 (8.7%) families carried this missense variant. In all four families, the *ACAD9* variant co-segregated with the pancreatic disease. However, up to four as yet unaffected family members below the age of 65 years also carried the variant. Since FPC develops at a median age of 65, these individuals were most likely too young to be affected yet. DNA from the paraffin-embedded corresponding tumor tissue from five affected PDAC individuals from three families was available for analysis. A mutation analysis of the *ACAD9* gene confirmed the same variant in the tumor tissue as that detected in the blood DNA.

## 4. Discussion

The genetic basis of familial pancreatic cancer is still not well defined. The genetic risk cannot be determined in over 80% of the families as the disease-causing gene is unknown. Therefore, in this study, germline whole-genome sequencing of 15 FPC families with at least 3 affected PDAC patients without identified *BRCA1/2*, *CDKN2A* and *PALB2* mutations was performed. As most high-penetrance disease-associated variants have thus far been identified in coding regions, we concentrated our data analysis on exonic variants [25]. The majority of identified potential predisposing genes had to be excluded, because their segregation with PDAC disease was not given. The presented results demonstrate that FPC is highly heterogeneous without a major predisposing gene defect. This finding is not surprising given prior genetic analyses of FPC [6,10,16,17]; however, there was the hope of identifying a previously undiscovered gene responsible for the majority of unresolved FPC cases. The presented data and also the WES data of 638 North American FPC patients [10] largely exclude this hypothesis, at least for truncating mutations within the coding regions of over 20,000 protein-coding genes.

The present WGS, however, detected potentially pathogenic variants in the *ATM*, *SUFU*, *DAB1*, *POLQ*, *MAP3K3*, *FGFBP3* and *ACAD9* genes that co-segregated with pancreatic disease in single FPC families, and thus might predispose them to the disease. This observation is in line with a recent North American WGS study that also reported deleterious low-frequency variants associated with FPC in some genes, such as *BUB1B*, *CPA1*, *FANCC* and *FANCG* [10]. In the present study, variants in these genes were either not detected (*BUB1B, FANCC*) or they did not co-segregate with PDAC in the 15 tested families (*CPA1, FANCG*). It has been shown, however, that other familial cancers, such as ovarian and colorectal cancer, are associated with co-segregating low-frequency (1–5%) and rare (<1%) genetic variants [26,27].

A new finding of the present study is that affected patients from one FPC family can carry identical germline variants in up to three different genes, e.g., *DAB1*, *POLQ* and *FGFBP3* in family 25-9-44 or *MAPK3* and *ACAD9* in family 25-4-46, which segregate with pancreatic disease. The legitimacy of this finding is supported by a recent study that reported deleterious germline variants in two or more analyzed genes, e.g., *ATM* and *PALB2*, in 32 FPC patients from independent families. However, segregation with the disease in individual families was not evaluated [10]. Notably, one family (25-5-67) in the present study, with an accumulation of PDAC and breast cancer, revealed a pathogenic *SUFU* variant co-segregating with PDAC and a *FANCM* variant segregating with breast cancer (Figure 2). This raises the question of whether alterations in more than one gene are required to develop a distinct phenotype in a PDAC-prone family. This could explain why some families with an identical segregating *BRCA2* mutation develop either predominantly PDAC or breast and ovarian cancer, and why some patients develop either one tumor type or even both [28,29,30].

Genome-wide sequencing identified a constitutional heterozygous *ATM* gene variant (W1795C) that co-segregated with PDAC in one of 15 tested FPC kindreds. No deleterious variants in the *ATM* gene were detected in another 21 FPC families, resulting in a prevalence of 2.8%. This prevalence lies within the reported range, between 2.2% (4/166) [31] and 5% (4/81) [32], from previous studies on FPC. These data suggest that *ATM* germline variants are among the most common variants found in FPC. However, their definitive role still remains unclear, since germline deleterious nonsense *ATM* mutations could not be detected in all affected members of an individual family in previous studies [10,33]. The ATM protein is a serine/threonine kinase involved in DNA double-strand break repair alongside several other PDAC susceptibility genes such as *BRCA1/2* and *PALB2* [34]. Despite its implications for counseling and screening, the identification of a pathogenic *ATM* mutation might, therefore, offer new therapeutic options for affected PDAC patients, such as treatment with the PARP inhibitor Olaparib, which led to a significantly improved progression-free survival in germline *BRCA1/2*-mutated PDAC patients after platinum-based induction [35]. Thus, the type of mutation can determine the choice of chemotherapy regimen in a more personalized manner.

A co-segregating, potentially pathogenic *SUFU* germline variant was detected in another family, which must be considered as a PDAC/breast cancer family. *SUFU* is a component of the Sonic Hedgehog signaling pathway, binds to and inhibits the Gli1 protein and loss of function mutations in *SUFU* and can cause abnormal constitutive upregulation of the downstream Gli-mediated transcription factors [36]. Aberrant Sonic Hedgehog signaling has been implicated in pancreatic tumor development, especially since its over-expression is observed in over 70% of PDACs and results in changes in the tumor microenvironment [37]. Germline mutations in *SUFU* are implicated in Gorlin syndrome and predisposition to childhood medulloblastoma [38]. However, a recent sequence analysis of 315 resected intraductal papillary mucinous neoplasms, which clearly belong to the phenotype of FPC [9,39], detected deleterious germline *SUFU* mutations in a few patients [40]. After Bonferroni correction for multiple tests, the authors found germline variants in the *SUFU* and *ATM* genes to be significantly enriched (*p* = ≤0.0001) among the patients compared to healthy controls [40]. In addition, *SUFU* shows somatic mutations in primary PDAC tissues [41]. Thus, *SUFU* might be an intriguing candidate for PDAC susceptibility, despite the fact that the *SUFU* variant showed incomplete penetrance with PDAC disease in the presented family, which is a known phenomenon of predisposing gene defects [42]. Large-cohort studies of IPMN and PDAC patients will, however, be needed to determine the prevalence of *SUFU* germline variants and their risk regarding tumor development. The same family carried a potentially deleterious *FANCM* variant, a gene involved in processes regulating DNA repair or chromosomal stability, as in PDAC and breast cancer susceptibility genes *BRCA2* and *PALB2*. The identified *FANCM* variant co-segregated with breast cancer rather than with PDAC in this family. This makes sense, since *FANCM* is a previously reported low-frequency gene associated with breast cancer [43].

A PCMS family (25-9-44) carried pathogenic low-frequency variants in the *DAB1, POLQ* and *FGFBP3* genes, with all three co-segregating with pancreatic disease. *DAB1* is a downstream mediator of the RELN pathway and plays an important role in the migration of neurons in the developing brain and in the formation of the cortical laminas [44]. Interestingly, the same *DAB1* (but somatic) splice mutation was found in the pancreatic cancer tissue of one FPC patient in a previous study [10]. In addition, the immunohistochemistry of normal and PDAC tumor tissue revealed the normal expression of DAB1 in the gallbladder and plasma cells, but a loss of expression in the PDAC lesions, suggesting a somatic second hit in the tumor tissue. This is in line with a study that postulated an association between the RELN pathway and the tumorigenesis of several different cancers, including PDAC [45].

The *POLQ* codes for a DNA polymerase are involved in double strand break repair. A *POLQ* germline variant of unknown significance, as in the presented family, has already been reported in non-*BRCA1/BRCA2*-mutated breast cancer families [46,47]. Other case–control studies also associated missense *POLQ* variants with an increased risk of breast cancer [48,49]. The presented data, however, suggest that low-frequency *POLQ* variants might also be involved in hereditary PDAC.

The third variant found in the *FGFBP3* gene in this family has not yet been associated with FPC. FGF-binding proteins (FGFBP) release fibroblast growth factors from the extracellular matrix storage and play a critical role as extracellular chaperones in the FGF-mediated signaling pathway and mitogenesis [50]. FGFBP expression is remarkably increased in PDAC compared with the normal pancreas and FGFBP3 was found to be induced early during pancreatic cancer carcinogenesis [51].

Another FPC family (25-4-46) carried a rare *MAP3K3* variant alongside an *ACAD9* variant. *MAP3K3* directly regulates the stress-activated protein kinase (SAPK) and extracellular signal-regulated protein kinase (ERK) pathways [52]. Thus far, it has been associated with breast cancer [53], esophageal cancer [54], colorectal cancer [55] and ovarian cancer [52].

For the first time, in this study, we identified an *ACAD9* variant (Ala326Thr) that was detected in every affected individual with PDAC or its precursor lesions in 4 of 46 (8.7%) FPC families that were characterized by the absence of breast cancer in the pedigree. According to a recent query of the GnomAD database, the allele frequency ranges from 0.0064 to 0.0132 (https://gnomad.broadinstitute.org/variant/3-128622922-G-A?dataset=gnomad_r2_1, accessed on 31 May 2020). The *ACAD9* gene, located on chromosome 3, is one member of the acyl-CoA dehydrogenase family and codes for a mitochondrial acyl-CoA dehydrogenase, family member 9, which is involved in the mitochondrial respiratory chain complex I and the oxidation of long-chain fatty acids. Heterozygous germline *ACAD9* mutations are associated with severe, isolated complex I deficiency and cardiac myopathy [56,57]. However, recent publications suggest possible roles for *ACAD9* in several cancers, such as prostate [58] and esophageal cancer [59]. In addition, Roberts et al. [10] reported a premature truncating variant in the *ACAD9* gene in one of 38 FPC kindreds (2.6%), among other more frequently mutated genes such as *BRCA2* and *ATM*. This underscores the potential importance of this gene in FPC. The exact mechanism of action of this variant is unknown at this time, but the fact that all four of the families carried the same germline variant might suggest that this *ACAD9* variant might act as an (proto-)oncogene.

## 5. Conclusions

In summary, the present study provides more evidence that the genetic basis of FPC is highly heterogeneous, without a major predisposing gene defect. In some families, the heritability and phenotype seem to be determined by the joint action of multiple low-frequency gene variants, and possibly also by their interaction with environmental factors. The findings regarding the co-segregating *SUFU, DAB1, POLQ, FGFBP3, MAP3K3* and *ACAD9* variants enlarge the list of potential FPC susceptibility genes. Further analyses of the function of these genes in biological pathways in future studies could shed light on their involvement in the development of pancreatic cancer, both sporadic as well as hereditary. The germline mutation status helps to predict the risk of neoplastic progression, allowing for a better risk stratification to determine the recommendations for the screening of individuals at risk. In addition, the type of mutation can determine the chemotherapy regimen that would be most successful for the individual using a more personalized approach.

## Figures and Tables

**Table 1:** Summary of major findings of whole-genome sequencing analyses in FPC families.

Gene	Family ID	Family Type	Variant	Mutation Type	Biological Significance (Pathogenic, VUS *)	Segregation with Disease	Families with the Variant	MAF	Somatic Gene Variant Described in PDAC Tumor Tissue
*ATM*	02-5-0382	FPC	c.5385G > T	missense	deleterious	yes	1/36	n.a.	no
			p.(W1795C)						
*SUFU*	25-5-67	FPC-breast	Position	promoter variant	pathogenic	yes	1/15	0.000024 ^1^	no
			−8C > T						
*DAB1* #	25-9-44	PCMS	c.786 + 1G > A	splice	pathogenic	yes	1/49	0.00001 ^1^	yes [7]
				site					
*POLQ* #	25-9-44	PCMS	c.4141C > A p.(P1381T)	missense	VUS	yes	1/29	0.015/	no
								0.025 ^2^	
*FGFBP3* #	25-9-44	PCMS	c.724G > A p.(A242T)	missense	deleterious	yes	1/29	0.000004 ^1^	no
*MAP3K3* §	25-4-46	FPC	c.1511T > C p.(I504T)	missense	deleterious	yes	1/29	0.00001 ^1^	no
*ACAD9* §	25-4-46; 25-1-91; 25-9-113; 25-2-209	FPC FPC FPC FPC	c.976G > A p.(A326T)	missense	VUS	yes	4/46	0.0152/ 0.0219 ^2^	no

*—VUS, variant of unknown significance; MAF, minor allele frequency; #—mutations were detected in the same family 44; §—variants were detected in the same family 46; FPC, familial pancreatic cancer; FPC—breast, pancreatic cancer/breast cancer; PCMS, pancreatic cancer-melanoma syndrome; ^1^—rare; ^2^—low.

## Data Availability

Patient data can be made available upon request.
